# Structural control of mixed ionic and electronic transport in conducting polymers

**DOI:** 10.1038/ncomms11287

**Published:** 2016-04-19

**Authors:** Jonathan Rivnay, Sahika Inal, Brian A. Collins, Michele Sessolo, Eleni Stavrinidou, Xenofon Strakosas, Christopher Tassone, Dean M. Delongchamp, George G. Malliaras

**Affiliations:** 1 Department of Bioelectronics, Ecole Nationale Supérieure des Mines, CMP-EMSE, MOC, Gardanne 13541, France; 2 Material Science and Engineering Division, National Institute of Standards and Technology (NIST), Gaithersburg, Maryland 20899, USA; 3 Department of Physics and Astronomy, Washington State University, Pullman, Washington 99164, USA; 4 Instituto de Ciencia Molecular, Universidad de Valencia, Paterna 46980, Spain; 5 Stanford Synchrotron Radiation Lightsource (SSRL), SLAC National Accelerator Laboratory, Menlo Park, California 94025, USA

## Abstract

Poly(3,4-ethylenedioxythiophene) doped with poly(styrenesulfonate), PEDOT:PSS, has been utilized for over two decades as a stable, solution-processable hole conductor. While its hole transport properties have been the subject of intense investigation, recent work has turned to PEDOT:PSS as a mixed ionic/electronic conductor in applications including bioelectronics, energy storage and management, and soft robotics. Conducting polymers can efficiently transport both holes and ions when sufficiently hydrated, however, little is known about the role of morphology on mixed conduction. Here, we show that bulk ionic and electronic mobilities are simultaneously affected by processing-induced changes in nano- and meso-scale structure in PEDOT:PSS films. We quantify domain composition, and find that domain purification on addition of dispersion co-solvents limits ion mobility, even while electronic conductivity improves. We show that an optimal morphology allows for the balanced ionic and electronic transport that is critical for prototypical mixed conductor devices. These findings may pave the way for the rational design of polymeric materials and processing routes to enhance devices reliant on mixed conduction.

## Abstract (web summary)

Conducting polymers are promising materials for applications including bioelectronics and soft robotics, but little is known about how morphology affects mixed conduction. Here, the authors show how bulk ionic/electronic transport is affected by changes in nano- and meso-scale structure in PEDOT:PSS films.

## Graphical abstract

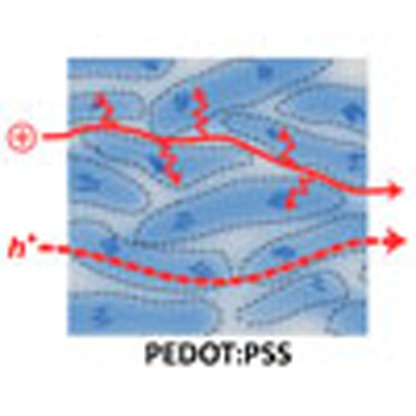

Mixed electronic/ionic transport in conducting polymers (CPs) is attracting a great deal of attention due to a host of new devices that leverage the coupling of these carriers to enable new modes of operation. One example is the organic electrochemical transistor (OECT), which consists of a CP layer in which ions injected from an electrolyte modify hole conductivity. Owing to their high transconductance^1^, these devices have been shown to be efficient transducers of biological activity, with applications ranging from neural interfacing^2^,^3^ to biosensing^4^. The inverse mode of operation, where a CP film injects ions into an electrolyte, has been used for drug delivery with exceptional spatio-temporal control^5^,^6^. In addition to applications in bioelectronics, OECTs are being developed for printed electronics^7^,^8^, and more recently, neuroinspired electronics^9^,^10^,^11^. Mixed transport in CPs is also leveraged in energy applications including batteries/supercapacitors^12^, electrochromic windows^13^ and in electromechanical actuators for soft robotics^14^,^15^. Despite the many opportunities enabled by mixed conduction in CPs, the lack of fundamental understanding of combined ionic/electronic transport and its connection to morphology hinders rational materials and device design.

A prototypical CP used in many mixed conductor devices is poly(3,4-ethylenedioxythiophene) doped with poly(styrene sulfonate); (PEDOT:PSS). This material has attracted interest for its high hole conductivity (>1,000 S cm^−1^), high stability and for its commercial availability as a dispersion for solution processing^16^. PEDOT:PSS dispersions are typically described as being composed of gel-like particles comprising a polyanion (PSS^−^)-rich shell which helps to stabilize the PEDOT-enriched particles in aqueous solvents^17^,^18^. PEDOT oligomers are believed to polymerize onto the PSS template (Fig. 1a), meaning that PEDOT itself is inherently ill-characterized as far as molecular weight and polydispersity. The gel particles (Fig. 1b) deposit in what has been described as a pancake-like morphology^19^,^20^,^21^, and show signs of *π*-stacking^22^,^23^,^24^,^25^ (Fig. 1c,d). PEDOT:PSS structure has been intensely studied by a variety of techniques including electron microscopy, X-ray scattering, elemental and surface-sensitive scanning probes^17^,^22^,^23^,^24^,^25^,^26^, largely with the goal of understanding and enhancing hole transport to make better electrodes for optoelectronic devices. Changes in formulation content, processing or post-processing have been shown to lead to enhancements in hole conductivity by inducing morphological re-arrangements, minimizing excess dopant phase (that is, insulating PSS) or both. A thorough review of the methods and suggested mechanisms to enhance conductivity can be found in ref. 27.

Ion transport in PEDOT:PSS (and in CPs, in general) has been explored only on a limited basis. The presence of electronic carriers in the film renders most techniques used to study ion conduction inutile. Nevertheless, the electrochromic nature of PEDOT:PSS, which gives rise to changes in film absorption on electrochemical (de)doping, allows us to follow ion motion in the film^28^,^29^,^30^, and to extract ion-drift mobility^31^. Similar to ion-exchange membranes such as Nafion^32^, hydration of PEDOT:PSS was found to establish ion-transport pathways, leading to a high ion mobility^31^. The nature of these ion pathways and their connection to film structure and morphology is not well-understood. Moreover, unlike the case of hole transport, attempts to modify the structure of PEDOT:PSS in a systematic manner and to monitor the effect on ion transport are lacking. It is important, for example, to deduce if hole and ion transport can be improved simultaneously, and, if not, whether an optimal morphology exists wherein device performance is maximized. This calls for a comprehensive study where both ion and hole transport are measured in films processed to have different structure and morphology. Thorough quantification of the latter is particularly important, as there have been differing reports on the influence of the same additive on film morphology^24^,^25^. This is a challenging proposition, as PEDOT and PSS have similar densities and bonding environments (limiting X-ray scattering contrast), and are disordered on multiple length scales.

Here, we investigate the microstructure and morphology of PEDOT:PSS films modified with varying amounts of co-solvent dispersion additive, ethylene glycol (EG), and relate these findings to measurements of electronic and ionic transport. To overcome the low contrast and a relatively disordered film microstructure, we use Synchrotron radiation and resonant soft X-ray scattering (rSoXS) and investigate the multi-scale microstructure of PEDOT:PSS films. The combined use of carbon-edge X-ray scattering and spectroscopy allows the deduction of the absolute nano-scale domain composition of the films. We find that the PEDOT:PSS-rich cores and PSS-rich matrix are surprisingly impure even with a large dispersion content of EG, but that the co-solvent enhances film heterogeneity. We then investigate both ionic and electronic (hole) transport in the same formulations. Ionic transport is probed using a spectroscopic analysis of the electrochromic moving front, revealing an ion motion heavily affected by the meso-scale domain size and composition. We find that ion and hole transport are oppositely affected by the addition of EG—a finding that is corroborated by the structural work herein. We show that this suggests an optimal morphology exists, enabling enhanced ionic mobility and hole conductivity, and leading to improved transconductance in OECTs. Therefore, this work presents a study of the bulk composition and microstructure of an organic electronic system, and its effect on mixed ionic and electronic transport.

## Results

### Structure in PEDOT:PSS films

The low-density contrast in PEDOT:PSS largely limits the utility of traditional electron or X-ray methods, however, the use of synchrotron radiation, as well as rSoXS allows for multi-scale structure determination. Wide-angle grazing incidence scattering (GIXS) of PEDOT:PSS films allows one to focus on the relative molecular packing arising from aggregation/crystallization^25^,^33^. Figure 2a and Supplementary Fig. 1 show the changes in wide-angle scattering on addition of EG as co-solvent to the PEDOT:PSS formulation. Dispersion co-solvents should not be present within the film (after film formation, annealing and vacuum treatment)^34^,^35^. *π*-stacking scattered intensity at *Q*=1.83 Å^−1^ is enhanced with higher EG formulation content. This enhancement is accompanied by a narrower peak width, indicating an increase in coherence length from ≅1 to 3 nm (due to larger crystallite/aggregate size, or a higher degree of aggregate ordering)^36^. A tendency for the *π*-stacked aggregates to slightly favour a face-on texture is observed, similar to previous reports^25^,^33^.

**Figure 1:**
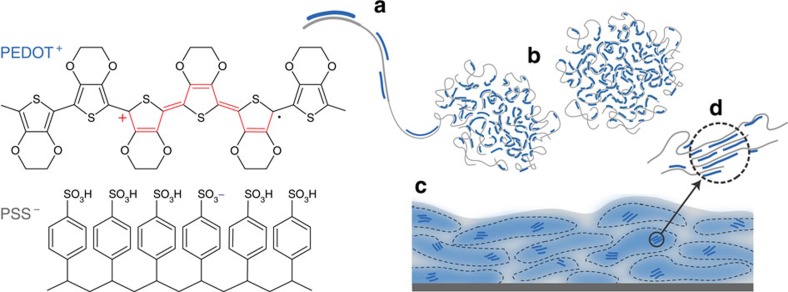
PEDOT:PSS structure and morphology. The chemical structure of PEDOT:PSS and commonly described microstructure of the CP system (**a**) synthesis onto PSS template, (**b**) formation of colloidal gel particles in dispersion and (**c**) resulting film with PEDOT:PSS-rich (blue) and PSS-rich (grey) phases. (**d**) Aggregates/crystallites support enhanced electronic transport.

**Figure 2:**
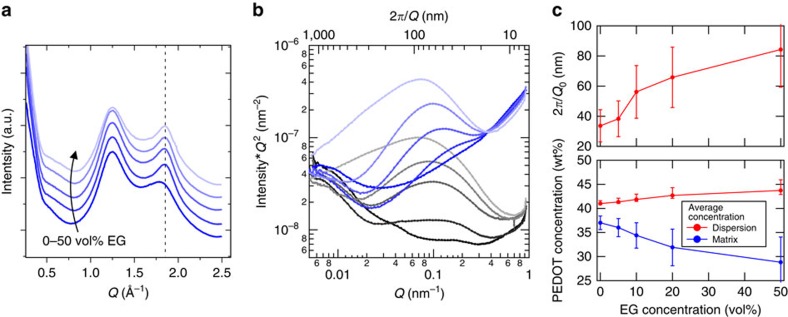
Microstructure and morphology of PEDOT:PSS from X-ray scattering. (**a**) Linearly offset wide-angle GIXS of PEDOT:PSS films cast from dispersions with EG concentration of 0, 5, 10, 20 and 50 vol% (varying from dark to light colours). The dotted vertical line is the position of the PEDOT:PSS *π*-stacking scattered intensity (020). (**b**) C-edge rSoXS of PEDOT:PSS films at pre-edge energy (270 eV, black) and at the C 1 s to π* resonance (285.1 eV, blue). (**c**) Domain spacing from peak position (2*π*/*Q*_0_) of the features observed at resonance in **b** and quantification of the absolute domain purity (as PEDOT concentration) from peak area. Uncertainties for purity are derived in Supplementary Fig. 3 (mainly from uncertainty in domain volume fraction), while uncertainties for spacing are calculated using the peak width to demonstrate the distribution of domain spacings.

Meso-scale structure of PEDOT:PSS films is investigated using C-edge rSoXS. The optical constants are determined from films of Na:PSS and PEDOT:Cl, which are dominated by the bonding of the polymeric/oligomeric species in the soft X-ray regime. The Na^+^ and Cl^−^ ions contribute a linear background absorbance but do not participate in the energetic transitions at the C-edge, enabling facile removal of their signals. The calculated materials and vacuum contrast functions can be computed^37^, and are shown in Supplementary Fig. 2. Two energies are selected for investigation, the pre-edge energy 270 eV, and the 1 s to π* C=C resonance at 285.1 eV. Figure 2b shows scattering profiles of PEDOT:PSS samples in the transmission geometry. The scattered intensity at *Q*<0.01 nm^−1^ (2*π*/*Q*>600 nm) matches well between the two incident energies tested for all samples measured, indicating a predominant contribution from vacuum contrast (that is, film roughness). The scattering feature in the *Q*≅0.1 nm^−1^ range is enhanced at resonance owing to materials (PEDOT to PSS) contrast (see Supplementary Fig. 2) and is thus a measure of the bulk meso-scale domain morphology. The upturn at the highest *Q*-values is primarily attributed to X-ray fluorescence (scaled by *Q*^*2*^), since it follows the energy dependence of absorbance and approaches a constant *I*(*Q*→∞). Some of the scattering at the highest *Q*-values may also originate from nanocrystals within PEDOT:PSS-rich domains. Previous works suggest the overall film morphology consists of closely packed PEDOT-rich domains in a PSS-rich matrix (Fig. 1c)^17^,^20^,^21^,^26^ thus, to quantify how the average meso-scale domain size and purity varies with EG, the features on a Lorentz plot *IQ*^2^(*Q*) are fit to a log-normal peak with a cubic background (Supplementary Fig. 3). The peak intensity (area) is found to scale linearly by one order of magnitude when EG content ranges from 0 to 50 vol%. This suggests a substantial increase in either domain purity or volume fraction, in concordance with the Porod Invariant formalism (see Supplementary Note 1 for specifics of the calculation)^38^. The feature position (Fig. 2c, top) is indicative of domain spacing (gel-like particle spacing in plane), and indicates the size of particles increases from 16 to 42 nm over the same range of EG concentrations, a size scale range in accordance with others^23^,^26^. Taken together, these data strongly suggest an increase of film heterogeneity with the addition of EG rather than having a homogenizing/inversion effect.

Compositional analysis using transmission NEXAFS of the same samples (same X-ray beam on the same spot as scattering) reveals only minimal variation in average film composition^39^, and corroborates the expectation that EG is effectively absent in the films^34^,^35^. The linear combination of the background-subtracted PEDOT:Cl and Na:PSS NEXAFS spectra is used to determine film thickness and composition. The PEDOT to PSS content in the film varies around 1:1.5 to 1:1.8 by weight, without a consistent trend with EG concentration, and is slightly lower than the as-received content of 1:2.5. Since there is not a large difference in the scattering population, the increased scattering intensity must arise from increased contrast between the scatterers. These findings confirm the domain purification on addition of the co-solvent, rather than loss of overall PSS content during the casting process.

Combining the NEXAFS and rSoXS analyses, we calculate an estimate of the absolute PEDOT concentration of the PEDOT:PSS-rich phase and surrounding PSS-rich matrix (Fig. 2c, bottom). The composition analysis (detailed in the Supplementary Figs 3–5) reveals that neither phase in the film is pure. As EG content in the dispersion is increased, the PEDOT concentration in the film's collapsed gel particles increases mildly from 41 to 44 wt%. Considering our X-ray diffraction evidence of PEDOT chain packing along the *π*-stacking direction, such a low PEDOT concentration suggests the aggregates that lie within these domains consist of alternating PEDOT and PSS chains along the non-*π*-stacking direction (similar to that of PEDOT:tosylate)^40^, rather than a crystal of pure PEDOT as suggested by others^24^. The PEDOT concentration varies more significantly in the matrix, where it decreases from 37 to 29 wt% with increasing EG co-solvent. These calculations assume a constant 0.75 volume fraction of the PEDOT enriched gel particles—an assumption based on cross-sectional AFMs reported by Nardes *et al*.^23^—which show a skin layer around the gel particles in this approximate volume ratio. The possibility of a changing volume ratio is included in the uncertainty calculations (along with other possible sources—see Supplementary Fig. 3). It is unlikely that the marked increase in scattering intensity, Δ*TSI*, originates from a change in domain volume fraction since our calculations show that such a Δ*TSI* would require an unrealistic volume fraction of >0.9. Indeed, with rSoXS results indicating a purification and rarefication of PEDOT: PSS-rich domains with the EG co-solvent, a pure skin layer covering these domains would be expected to pose a significant barrier to hole conductivity. Although our measurements cannot address connectivity directly, the calculated PEDOT concentration in this skin layer above 20 wt% indicates a possible means for percolation of charges between the PEDOT:PSS-rich particles.

To the best of our knowledge, this is the first time an analysis of absolute nano-scale domain composition in an all-polymer blend has been achieved using a combination of NEXAFS and X-ray scattering at a resonant photon energy of a molecule. It should be noted that the resonant scattering experiments described above are performed in transmission, with the incident X-ray beam normal to the plane of the film. Therefore, only the lateral meso-scale structure is quantified. Previous works show the collapsed gel particles (the pancake-like morphology) possess an approximate aspect ratio of 0.1–0.5 (ref. 41), with the out-of-plane direction possessing a *Q*-value out of the range of the current experimental setup. Despite this known anisotropic morphology, the trends in domain composition should not be significantly affected, and are discussed in detail in Supplementary Fig. 4.

### Ionic transport in PEDOT:PSS

**Figure 3:**
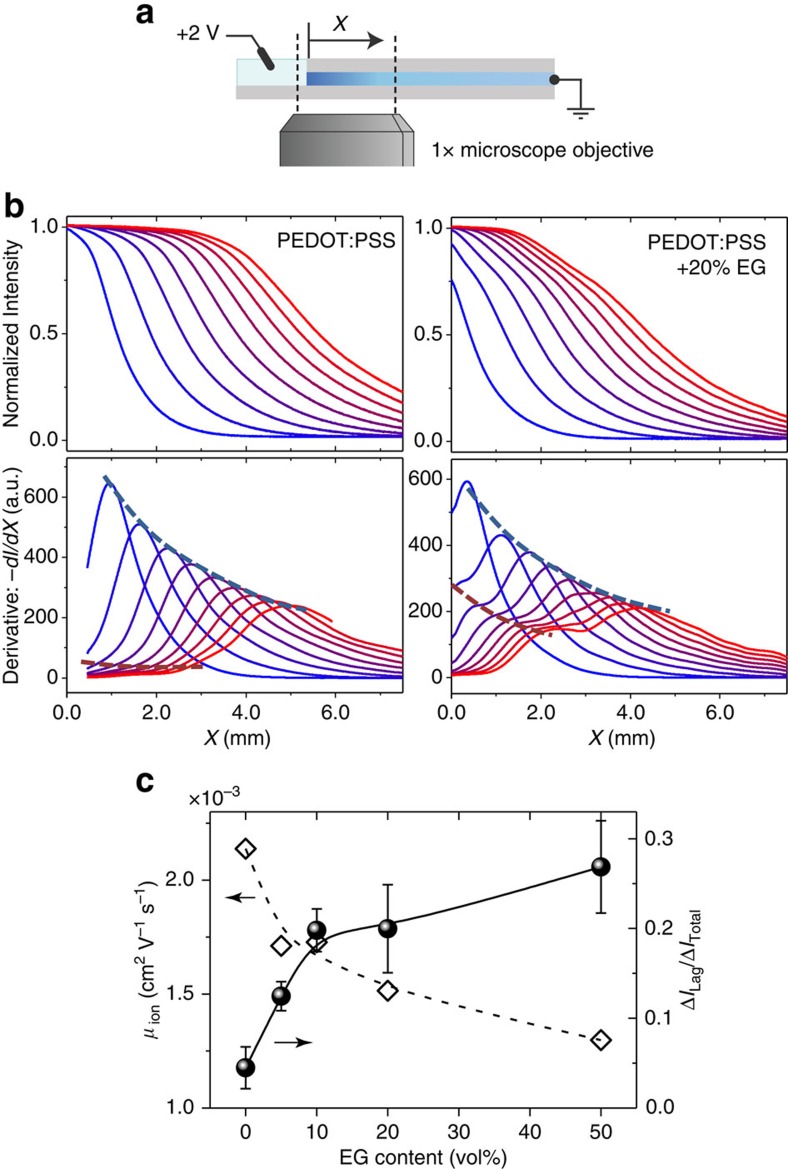
Co-solvent influences ion transport. (**a**) Temporal characteristics of spatial moving front profiles due to ion transport. (**b**) 1D normalized-intensity profiles as a function of distance, X, from the electrolyte-PEDOT:PSS interface (from *t=*5 s, blue, to *t=*45 s, red; Δ*t=*5 s) are obtained from time-lapse optical micrographs. Use of EG as a co-solvent affects the shape of the moving front profiles, which is highlighted by investigating the spatial derivative of the intensity profiles, revealing a ‘leading' and ‘lagging' front (blue and red dotted lines, respectively). (**c**) The effective ion mobility as derived from the moving front data (open symbols/dotted line), and the fractional contribution of the lagging front (Δ*I*_Lag_/Δ*I*_Total_) (solid symbols/line), as a function of the EG content in the dispersion. Error bars were determined from the standard deviation of Δ*I*_Lag_/Δ*I*_Total_ from 5–10 consecutive spatial derivative profiles in the *t=*5–50 s range (the inclusion of a specific profile in quantification depended on the ability to fit both leading and lagging contributions within the experimentally restricted spatial range, *X*).

We probed ion transport in PEDOT:PSS films with a one-dimensional version of the ‘moving front' experiment^31^. The method involves monitoring visible light transmission through an electrochromic film as it is (de)doped due to lateral injection of ions from a planar junction with an electrolyte (Fig. 3a). The drift mobility of ions is then determined from the time-dependent position of the moving front, by assuming a simple equivalent circuit model^42^. Applying this methodology to our EG-containing PEDOT:PSS formulations allows us to relate the meso-scale structure of films, discussed above, with ion motion therein.

The electrochromic moving front is represented as a 1D normalized-intensity profile at each time point (Fig. 3b, top and Supplementary Fig. 6). The effective mobility of K^+^ ions, *μ*_ion_, drops consistently and in a reproducible manner from 2.2 × 10^−3^ cm^2^ V^−1^ s^−1^ to 1.3 × 10^−3^ cm^2^ V^−1^ s^−1^ for films prepared with 0 and 50 vol% EG, respectively (Fig. 3c). The EG-induced reduction in effective ionic mobility is most likely due to the changes in meso-scale arrangements.

The subtle change in the shape of the moving front profiles on addition of EG reveals that the ion motion can be described by two moving fronts. This behaviour is more clearly observed in the derivative of the front profiles (Fig. 3b, bottom and Supplementary Fig. 6, bottom). The derivative profiles can be used to deconvolute the moving front into a ‘leading' front and a ‘lagging' front (Supplementary Fig. 7), whereby the fraction of PEDOT:PSS de-doping attributed to each wave of effective ion motion is deduced. With 50 vol% EG, up to 30% of the complete film de-doping is due to the lagging front alone—a contribution which is negligible when no co-solvent is used (Fig. 3c). The evolution of the lagging front is correlated with the position and breadth of the leading front, ruling out the possibility of different (impurity) cations.

To further deduce the origin of different moving fronts observed in EG-modified films, we add an element of absorption spectroscopy to the lateral moving front experiment and monitor the time-resolved ion motion through the local microstructure. When a cation effectively de-dopes a PEDOT chromophore, the associated change in the visible absorption spectrum of the film can reveal not only the presence of ions, but also the local molecular order of the chromophore in question. The higher energy feature of a polythiophene absorption spectrum is typically associated with amorphous molecular arrangements, as in solution phase, while the low-energy vibronic progression is attributed to weakly interacting H- and J-aggregates^43^. The intensity and ratios of the vibronic peak progression are correlated with the degree of local order of the conjugated backbone^44^.

**Figure 4:**
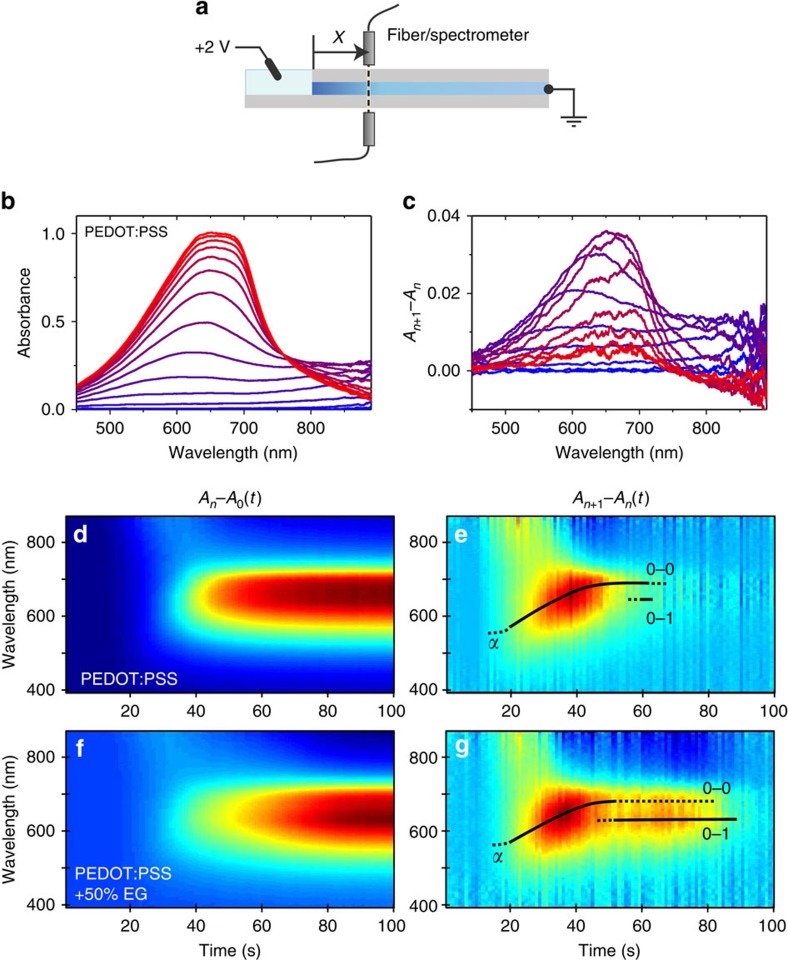
Spectro-temporal characteristics of ion transport and the effect of EG content. (**a**) Spectro-temporal data are collected with a white-light fibre spectrometer at a fixed position (*X*≅4 mm) from the edge of the well. (**b**) Absorbance data (*A*_n_−*A*_0_, where the subscripts represent time *t*=*n* and *t*=0 s) of a hydrated PEDOT:PSS film (0 vol% EG) as the de-doping front moves past the position of the optical fibre (*t=*0 s, blue to *t=*60 s, red). (**c**) Temporal derivative spectra (*A*_n+1_−*A*_n_) of the same film revealing the variable electrochromic signatures associated with K^+^ transport as the moving front passes. (**d**,**e**) Colour plot representations of the data in **b**,**c**. (**f**,**g**) Colour plot spectra of PEDOT:PSS with 50 vol% EG in the formulation. Note that the time to reach saturation increases with EG formulation content; the added time is due to a lengthy tail of aggregate absorption, as noted by the 0–0 and 0–1 vibronic features (**e**,**g**).

A fibre light source/spectrometer is mounted at a distance of *X*≅4 mm from the edge of the CP film/electrolyte interface, in the same configuration as that of the microscopy measurements (Fig. 4a). The ultraviolet–visible spectra are recorded in 1s intervals as a +2 V bias is applied across the film (Fig. 4b,d,f). To deduce the time course of the instantaneous optical signature and to better understand the local ionic environment, we plot the absorption of individual 1 s intervals (Fig. 4c,e,g). For all samples, we observe first the arrival of the front at *t*≅10 s, after which the spectra evolve from a broad peak absorption at ∼550–600 nm, to A_0–1_ and A_0–0_ vibronic features at 625 and 685 nm, respectively, over a 40–50 s time span. The sustained addition of vibronic absorption persists in EG-rich samples (Fig. 4g) for an additional 30–40 s (for 50 vol% EG), and is responsible for the delay in reaching saturation in Fig. 4f. The spectroscopy data thus suggest that the rapidly moving leading front is due to ions passing through regions of disordered PEDOT chromophores (most likely those in the PSS-rich matrix), while the lagging front is dictated by ion penetration into aggregate-rich domains such as those in the PEDOT:PSS-densified cores. The persistence of the aggregate absorption recovery within films cast from a higher EG content directly relates to the existence of a lagging front, and thus suggests that aggregates and domain content dictate effective ion mobility.

### Implications for device performance

**Figure 5:**
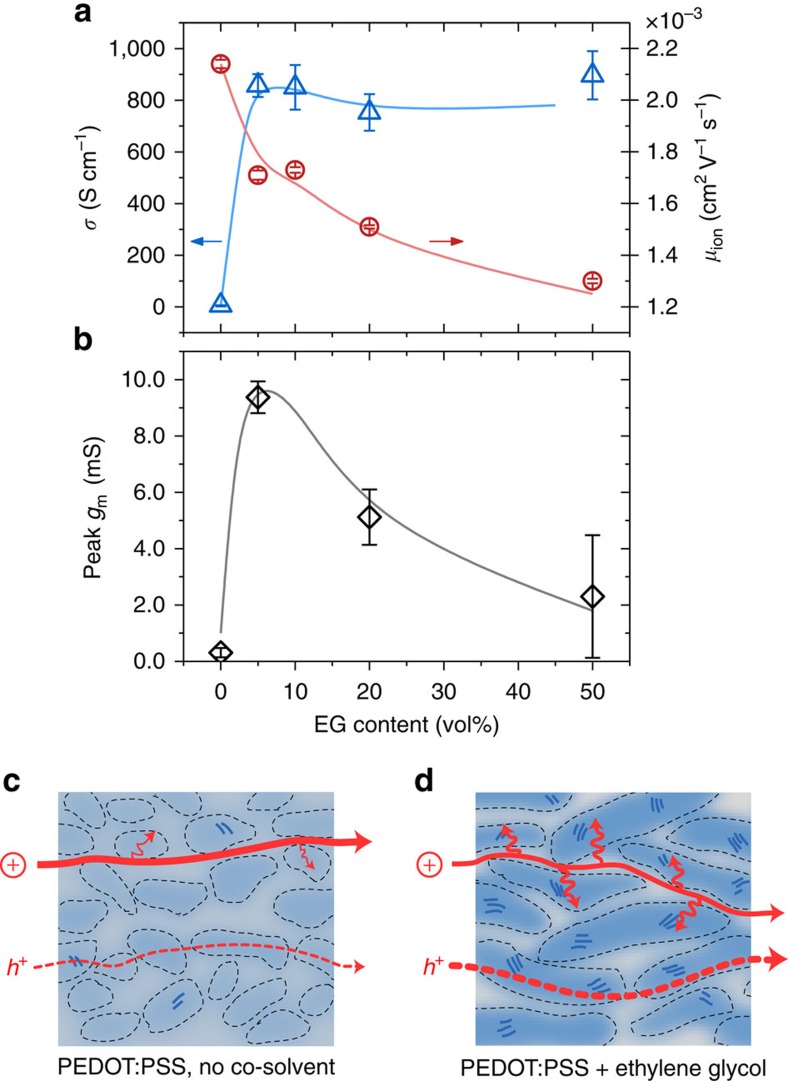
Effect of mixed conduction in PEDOT:PSS on OECT performance. (**a**) Electronic conductivity (blue), K^+^ ion mobility (red) and (**b**) OECT peak transconductance (*g*_m_) at *V*_*D*_=−0.6 V as a function of EG formulation content. Devices have dimensions *W/L=*50 μm/50 μm and an equivalent thickness of *d*=200 nm. Solid lines are guides to the eye. Note that neither efficient electronic nor ionic transport alone is sufficient for achieving the highest *g*_m_. A schematic summary of the morphological changes and associated transport of ions and holes viewed in the cross section of bulk film of PEDOT:PSS (**c**), and PEDOT:PSS with EG (**d**). Addition of EG varies the PEDOT content (shade of blue), size/purity of PEDOT:PSS-rich phases, and amount of PEDOT:PSS aggregates/crystallites. Error bars in **a** represent standard deviations of conductivity values from *n*=8 measurements, while those in **b** are from OECT measurements of four transistors for each EG condition.

Figure 5a shows that the conductivity improves from 6 to 800 S cm^−1^ upon addition of just 5 vol% EG, in agreement with previous reports^45^,^46^. Increasing the EG content >10 vol% does not lead to a notable change in conductivity of the films. The trends in effective ion mobility in films of the same formulation are shown overlayed in Fig. 5a. The film cast from PEDOT:PSS dispersion without EG displays maximum ionic mobility but poor electrical conductivity, while on addition of 50 vol% EG to the dispersion, the electrical conductivity is maximized at the expense of ionic mobility. Figure 5b shows that neither of these cases is optimal, but it is the 5 vol% EG formulation which yields OECTs with the highest transconductance (*g*_m_) (Supplementary Fig. 8). The formulation with 0 vol% EG leads to an ≅30 × lower *g*_m_ despite showing the best ionic mobility, and the one with 50 vol% EG shows ≅4 × lower *g*_m_ despite displaying the highest electrical conductivity (Fig. 5b).

The difference in effective ionic mobility between samples with low (5 vol%) and high (50 vol%) EG content may seem small in comparison with the observed variation in OECT transconductance. The ion mobilities determined from the moving front displacement are, in fact, dominated by lateral drift of ions, whereas operation of the OECT implies initial through-plane penetration of ions, such that a direct correlation of ionic mobility to device performance is not straightforward. Especially for the films prepared from the dispersions of high EG content, the fractional contribution of the lagging front on the total moving front increases substantially (see Fig. 3c). Furthermore, due to the larger lateral extent of domains, these films may show a higher degree of anisotropy, with more tortuous pathways in the out-of-plane direction through the PSS-rich regions. Taken together, ion mobilities determined from lateral moving front experiments, in particular for the films cast from high-EG-fraction dispersions, are likely overestimations of the through-plane ion transport.

## Discussion

Correlating device performance with transport and ultimately film microstructure/morphology is the primary route by which to design new formulations and materials. Addition of the higher boiling point polar solvent, EG, in the casting dispersion leads to changes in the film structure at both the molecular and meso-scale (see Fig. 5c,d). EG leads to an increase in PEDOT aggregation, and slightly tighter *π*-stacking, in agreement with the work of Palumbiny *et al*.^25^,^33^ At the same time, the domain size of the gel-like particles is known to grow^26^,^47^, increasing by a factor of 3 in the in-plane direction in this work. The growth of domain size with higher EG content is accompanied by a purification of the PEDOT:PSS-rich cores (towards the ultimate limit of 1:1 PEDOT:PSS), and of the PSS matrix (∼30% reduction in PEDOT; a lower purity than that observed in drop casting studies)^48^.

The coarsened, heterogeneous film morphology of films cast with EG is responsible for the observed dual moving front profiles in Fig. 3b, and leads to lower effective ion mobility. The spectroscopic investigations show that films cast from formulations with higher EG content exhibit a longer, lagging tail of visible absorption recovery (de-doping) attributed to the interaction of ions with aggregated PEDOT chromophores. This aggregate absorption is responsible for the enhanced contribution of the lagging front in the 50 vol% EG film—up to 27% of the total extent of de-doping. We propose, therefore, that the transport of ions takes place through the PSS-rich regions (via the initial leading front), but that the effective ionic mobility is limited by the subsequent diffusion of ions into the densified PEDOT:PSS-rich domains (which slows the total de-doping front).

The observed improvement of hole transport and deterioration of ion transport on the addition of EG thus agree with changes in the multi-scale morphology. At the inter-molecular level, the quality of hopping between molecules is aided by tighter *π*-stacking and enhanced aggregation^33^, largely improving electronic conductivity via an enhancement in hole mobility. At the meso-scale, the growth and purification of PEDOT:PSS-rich domains should aide in electronic transport, however, the percolative nature of the network—the connectivity of the collapsed gel-like particles—cannot be probed with the scattering techniques used. The increase in PEDOT content in the cores, due to potential recruitment of previously inactive PEDOT into the electrically percolating network, may serve to further enhance the film conductivity by increasing hole concentration. Ultimately, the same *π*–*π* aggregation and densification of the PEDOT:PSS cores that aides in improving electronic transport, slows down the effective ion motion, leading to opposite trends in conductivity and ion mobility.

The trend in OECT performance, and its similarity to the convoluted trends in electronic and ionic transport give credence to the importance of mixed conduction for device operation. OECT operation relies on efficient lateral bulk electronic transport, and good vertical ion penetration into the film. As can be seen here, these requirements are often contradictory with electron transport being supported by rigid backbones with *π*-stacked crystallites, while ion transport would benefit from an open, hydrogel-like morphology. Balancing these needs will lead to efficient mixed conduction-based devices. It has been shown that the product of electronic charge carrier mobility (*μ*), and the volumetric capacitance (C*) is the material's figure-of-merit for high transconductance devices^3^. Indeed, a high C* requires that hydrated ions readily access the largest possible percolating conjugated polymer (PEDOT) content, placing greater importance on the lagging front (ions arriving at PEDOT aggregates).

OECT device operation, especially for applications in bioelectronics, necessitates an aqueous environment. Inherently, some of the techniques used here are limited in their ability to probe the films in their fully hydrated state. X-ray scattering and conductivity measurements were performed in the dry state, while the films were in contact with aqueous media during the moving front experiments, the latter most resembling device operation. While film hydration may affect the meso-scale spacings/relative domain sizes, the transport trends observed with EG formulation content are preserved. This verification was performed by extracting the hole mobility of PEDOT:PSS films from OECT operation (see the ‘Methods' section) for different EG casting conditions, and comparing the trend with the conductivity/mobility obtained for dry films (Supplementary Fig. 9). Hydrated films were found to have a slightly higher mobility, with larger discrepancy noted for the pristine PEDOT:PSS films. Estimates of hole mobility from Hall effect measurements in this work, for example, *μ*=1.3 cm^2^ V^−1^ s^−1^ for the 20 vol% EG-cast film, are in agreement with previous reports^49^, and are on par with OECT-determined hole mobility for the same formulation (*μ*=2.7 cm^2^ V^−1^ s^−1^). This result is in agreement with Wang *et al*.^50^ who reported that relative humidity affects the electronic conductivity of PEDOT-based films only when the ionic transport governs the electrical properties. Accordingly, environmental humidity is crucial for poorly electrically conducting films, such as melanin, where conductivity is dominated by proton hopping^51^. Taken together, the agreement of mobility between dry and hydrated PEDOT:PSS films, and the conservation of the general trends observed, confirm that structure–electronic property correlations can be made for the present system independently of the polymer hydration.

By revealing the multi-scale morphological changes triggered by the addition of a commonly used co-solvent, we are able to establish structure-property relations describing mixed conduction in a prototypical CP. We extend the state of understanding of PEDOT:PSS film structure by quantifying domain composition using NEXAFS and resonant scattering techniques. Even with a large volume fraction of EG in the casting dispersion, the phases are seen to trend towards purification, with the PSS phase being far from pure. The observed phase purification enhances electrical conductivity while adversely affecting effective ion mobility as observed in electrochromic moving front studies. Here, the co-solvent EG is used as a means to tune both electronic and ionic transport, and to maximize the performance of OECTs. Finding routes to simultaneously improve ionic and electronic conductivity of CPs and their devices is relevant for many rising materials for applications in bioelectronics, electronics, energy storage and management, and robotics. Enhancing ion transport by approaching hydrogel-like structures without limiting long-range electronic transport remains a materials design hurdle in CP systems. Nevertheless, the structure-mixed conduction property work presented here lays the foundation for future materials design of organic mixed conductors.

## Methods

### Materials and film formation

Films for structural, electronic and planar junction ion-transport measurements were spun from aqueous dispersion formulation onto glass, Si or Si/SiN window substrates (cleaned by ultra-sonication in dilute soap and acetone/IPA, and/or rinsed in DI water before drying). Substrates are cleaned for 2 min in oxygen plasma before spin casting the dispersions: PEDOT:PSS (Clevios PH-1000 from Heraeus Holding, GmbH) with dodecyl benzene sulfonic acid (DBSA; 0.002 vol%) is mixed with EG (between 0–50% by volume of dispersion as noted in the text/figures) before ultra-sonication (10–15 min) and filtration (through 1.2 μm cellulose acetate filters). Unless otherwise noted, all chemicals are purchased from Sigma Aldrich. Formulations are spun at 1,000–3,000 r.p.m., 1–3 times, soft baked after each spin (90 °C, 60 s), followed by a hard bake (140 °C, 40 min).

### X-ray scattering

Grazing incidence wide-angle X-ray scattering was carried out on films spun on native oxide silicon and was performed at the Stanford Synchrotron Radiation Lightsource (SSRL) on beamline 11-3 (2D scattering with an area detector, MAR345 image plate, at grazing incidence), with incident energy of 12.7 keV. The incidence angle was slightly larger than the critical angle, ensuring that we sampled the full film depth, and measurements were performed in a helium-filled chamber to minimize beam damage and reduce air scattering. Data are expressed as a function of the scattering vector *Q=4π*sin*(θ)/λ*, where *θ* is half the scattering angle and *λ* is the wavelength of the incident radiation. Wide-angle GIXS data were distortion corrected and integrated from *χ*=0 to 90°, where *χ* is defined as the angle between the orientation of a scatterer and the surface normal. Films for rSoXS were solution cast onto 1.5 mm × 1.5 mm, 75 nm thick Si_3_N_4_ membranes (Norcada, Inc.). Scattering measurements were acquired in transmission geometry onto a CCD at beamline 11.0.1.2 of the Advanced Light Source (ALS, Lawrence Berkeley National Lab) using previously published methods^52^.

### Near-edge absorption fine structure

NEXAFS spectra for optical constants were measured in transmission mode via a phosphor-coated PMT in a scanning transmission X-ray microscope at beamline 5.3.2.2 of the ALS. The PEDOT:Cl (as in ref. 53) and Na:PSS (from Polymer Souce, spin cast from 1 mg ml^−1^ solution) films were cast onto Si_3_N_4_ membranes. The measurement and data processing followed previously published methods^37^. NEXAFS spectra for average film composition were measured at beamline 11.0.1.2 of the ALS in transmission mode using a photodiode and illuminating the same film spot under investigation for scattering. NEXAFS measurements before and after the scattering experiment were used to confirm no sample damage occurred during the measurement.

### Moving front characterization

Planar junctions for electrochromic moving front experiments were prepared as described previously^31^. Briefly, The PEDOT:PSS formulations described above are spun cast on 2-μm thick parylene-C (SCS Labcoater 2) on a 25 × 75 mm glass substrate, before vapour depositing a gold electrode on one side of the film. A≅40-μm-thick SU-8 film was spin-coated, patterned and baked on top of the PEDOT:PSS film to serve as an ion barrier. A polydimethylsiloxane rim was placed on top of the SU-8 well to confine 1.5–2 ml of electrolyte. The length of PEDOT:PSS film between the electrolyte and the Au contact was 3.2 cm. A Ag/AgCl electrode (Warner Instruments) was immersed in the 100 mM KCl in DI water electrolyte. De-doping was achieved by applying a +2 V bias to the Ag/AgCl electrode; the device was then short-circuited to return to its doped state (this defined one cycle). Measurements were reproducible after 2–3 cycles. Spatio-temporal measurements were conducted on an inverted Carl Zeiss Axio-Observer Z1 in the bright-field mode with a 1 × objective. Voltage was applied, and current measured using a Keithley 2612A source-measure unit connected to a computer running custom LabView software. Grey-level profiles, front shape normalization and deconvolution were calculated using a custom Matlab program. UV-visible spectro-temporal measurements were carried out by mounting the planar junction devices in a 3D-printed holder with fibres for the light source (Hamamatsu) and detector (Ocean Optics USB4000) aligned across from each other at a fixed *X* distance from the edge of the well. Time-lapse spectra were taken every second using Ocean Optics OceanView software. *A*_n_−*A*_0_, *A*_n+1_−*A*_n_ spectra, normalization and spectral-time colour plots were obtained through custom Matlab software.

### Electrical characterization

Conductivity of the polymer thin films were measured with a Van der Pauw Ecopia HMS-3000 Hall Measurement System. PEDOT:PSS formulations with increasing EG concentrations were coated on 10 × 10 mm^2^ glass slides with patterned ITO contacts on the corners. IV scans confirmed the ohmic behaviour of the sample contacts. Measurements were performed at room temperature using a current source of 1 mA and a magnetic field strength of 0.56 T.

OECTs were fabricated in a cleanroom and patterned photolithographically, as previously described^1^. They consisted of a PEDOT:PSS channel (formulation described above, with 0.1 wt% of (3-Glycidyloxypropyl)trimethoxysilane to stabilize the film in aqueous) with Au source and drain electrodes and interconnect lines. Parylene-C was used to insulate the Au interconnects from the electrolyte. A Ag/AgCl pellet (Warner Instruments) was used as the gate electrode. Electrical measurements followed previously defined protocols^1^. Device characterization/analysis were carried out with custom LabView and MATLAB software. Hole mobilities from working OECTs were determined using drain current transients at different constant gate currents, which allows for extraction of hole transit times. The approximate electric field at a low drain bias along with the hole velocity allows for mobility estimation, as shown in refs 3 and 54.

## Additional information

**How to cite this article:** Rivnay, J. *et al*. Structural control of mixed ionic and electronic transport in conducting polymers. *Nat. Commun.* 7:11287 doi: 10.1038/ncomms11287 (2016).

## Supplementary Material

Supplementary Information Supplementary Figures 1-9 and Supplementary Note 1.
