# Supplementary material for: Structural control of mixed ionic and electronic transport in conducting polymers
Source: Nat Commun. 2016 Apr 19;7:11287. doi: 10.1038/ncomms11287 (PMC4838877; doi:10.1038/ncomms11287)
Supplement: Supplementary Information — Supplementary Figures 1-9 and Supplementary Note 1. [file ncomms11287-s1.pdf]

Supplementary Figures

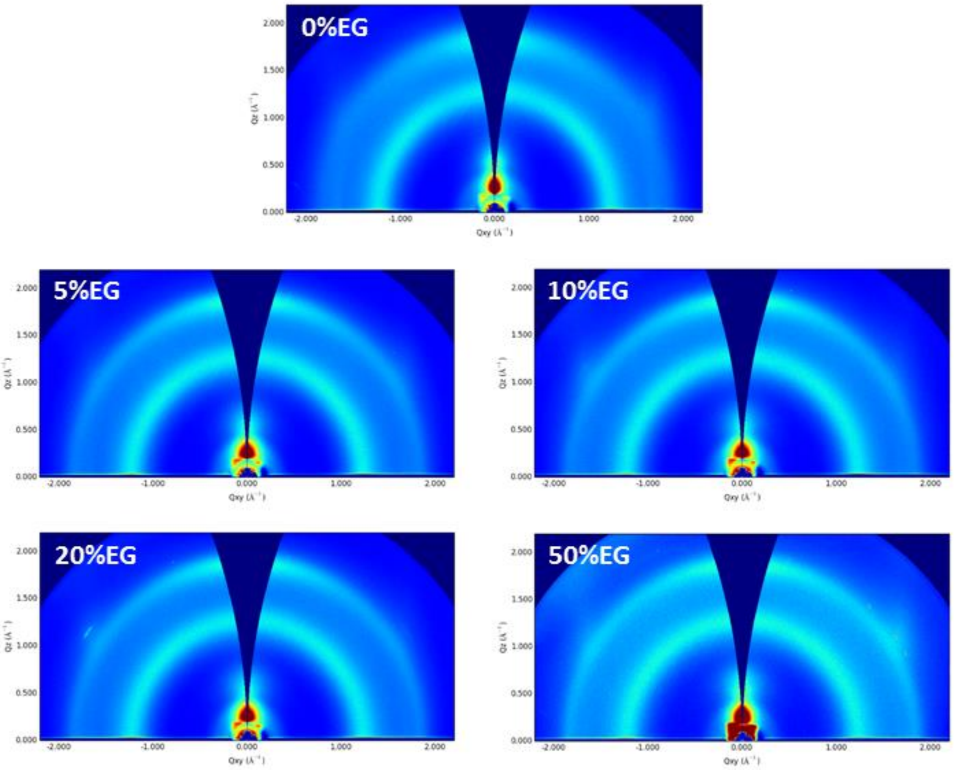

Supplementary Figure 1. 2D-GIWAXS of PEDOT:PSS with varying EG content in dispersion.

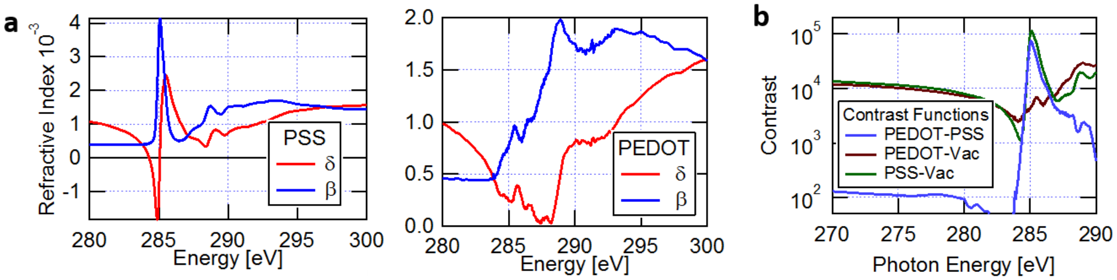

Supplementary Figure 2. Contrast of PEDOT to PSS. a. Optical constants (*n*=1-*δ*+*iβ*) for Na:PSS (left), and PEDOT:Cl from NEXAFS. **b**. Calculated scattering contrast ($\Delta n_{a,b}^2$) between materials and vacuum and between the two materials (PEDOT and PSS).

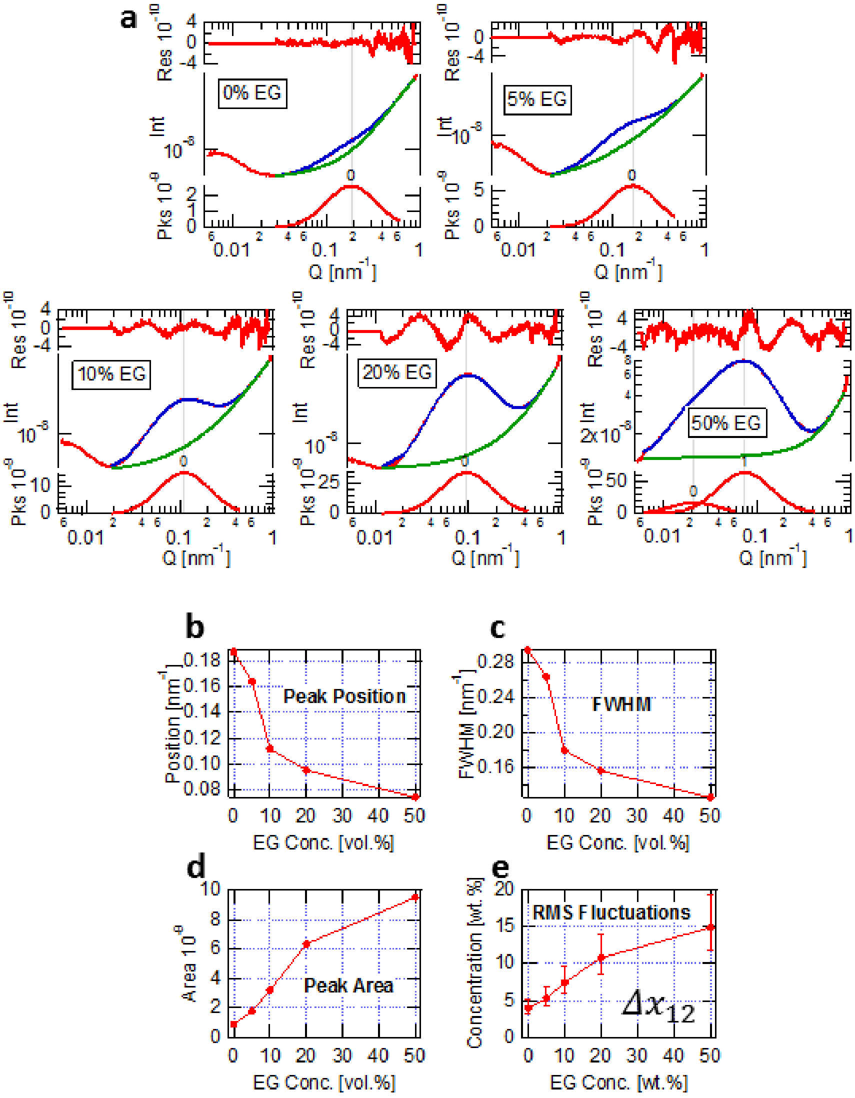

Supplementary Figure 3. Fitting routine for rSoXS parameter extraction/analysis. a. Peak fits to rSoXS of each sample using a log-normal peak (below data in red) on a cubic background (green) to approximate the mesoscale scattering intensities. Fit curves are blue overlaying the red data. **b, c, d.** Extracted fit parameters from the above fits: peak position (b.), FWHM (c.) and peak area (d.). **e.** RMS compositional fluctuation in the film (Δx_{12}) assuming a constant volume fraction of the PEDOT-rich particles of 75%. Calculated uncertainties are from the unknown volume fraction (0.5 to 0.85), detector calibration (±15%), and possible non-spherical symmetry of the scattering due to film thickness (±15% - see Figure 4).

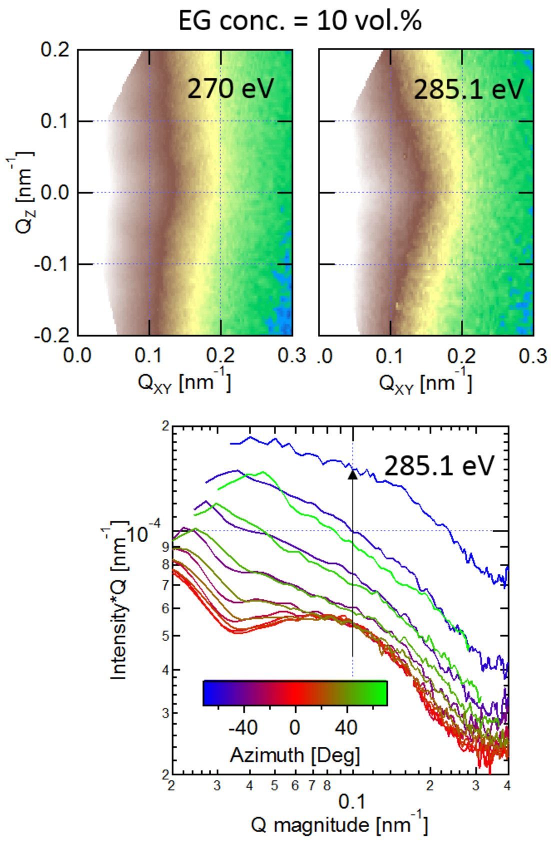

Supplementary Figure 4. Confirming spherical scattering symmetry. $I(Q_Z, Q_{XY})$ was measured by tilting the sample in the beam. The resulting 2D scattering maps (top) show scattering largely dominated by the film termination rod, especially at 270 eV. However, at 285.1 eV, a bulge in the scattering intensity reveals the domain scattering. Polar profiles of the scattering intensity (defined as $\phi = \pm 90°$ at the poles) were extracted (bottom) which reveal the scattering feature displayed in Figure 2 in the main text. This feature doesn't change or shift significantly as a function of ϕ, although it becomes increasingly difficult to confirm this at the highest angles ($\phi > 60°$) due to the background from the film termination rod. From these results, we conclude that our spherical scattering symmetry approximation in our TSI calculation is accurate. It is worth noting that, in reality, we expect a difference in structure in the pure Q_Z direction due to the collapsed gel particle shape of the PEDOT domains. However, an integral accounting for a polar angle-dependence in intensity will preferentially weight angles between ± 60 ° due to the $\cos(\phi)$ Jacobian. Thus, deviations from spherical symmetry near the poles are not very important for the TSI calculation.

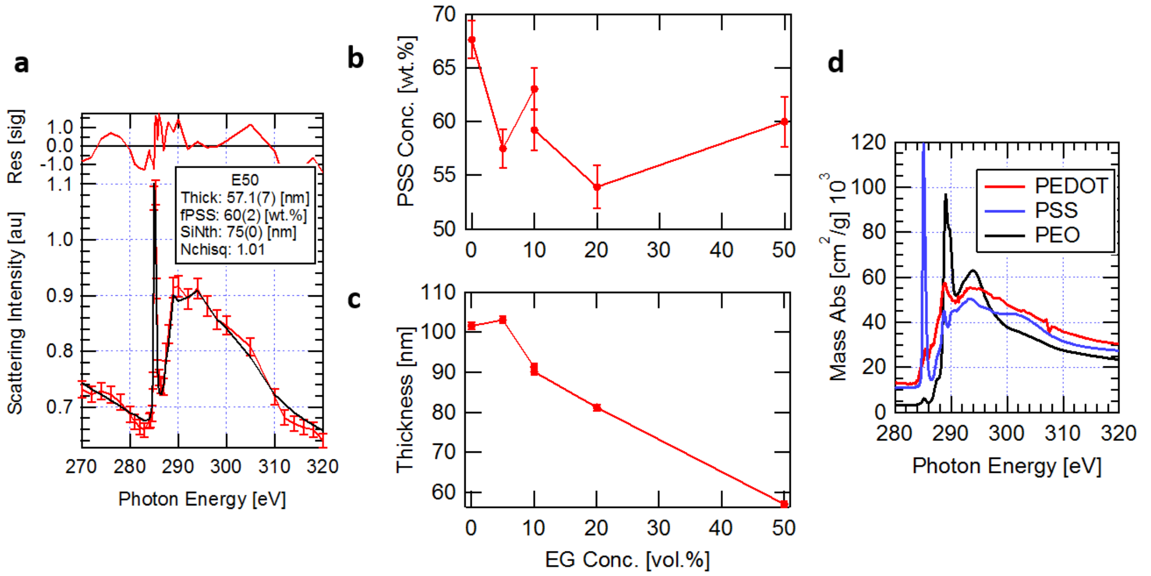

Supplementary Figure 5. PEDOT:PSS weight fraction and film thickness from fitting transmission NEXAFS spectra using the pristine Na:PSS and PEDOT:Cl spectra in Supplementary Figure 2, assuming 75nm thick SiN*x* window thickness. **a.** An example fit to a film cast using 50 vol.% EG co-solvent. The two-parameter fit employs the pure PEDOT and PSS spectra in a linear combination. The excellent reproduction of the data with the model and resulting reduced $\chi^2 = 1$ with only two parameters indicate good accuracy of the values extracted and lay credence to the absence of any remaining EG in the film. **b.** PEDOT:PSS with EG varies from 66 wt% PSS (1:1.9 PEDOT:PSS) in the film without EG, to 60 wt% (1:1.5 PEDOT:PSS) for the film processes with 50% EG. **c.** Film thickness decreases gradually from 103 nm to 58 nm as EG content is increased, likely from the lower relative content of PH1000 dispersion in the ink. **d.** Reference NEXAFS spectra used in the fits. Poly(ethylene oxide) NEXAFS is used to simulate the spectral uniqueness of the ethylene glycol co-solvent. In particular, if the co-solvent remained in the film, there would be an unbalanced spectral weight of the oxygen bond at 289 eV and hydrogen bond at 293 eV as well as a lower pre-edge due to the lower concentration of sulfur. Thus, in this scenario, the low reduced chi fit obtained for the PEDOT:PSS films would not be possible. Error bars in (a) are from the counting statistics (shot noise) in the NEXAFS measurement, errors in (b,c) are from the confidence in the two component fit of the pristine NEXAFS spectra to the spectra of films with varying EG content.

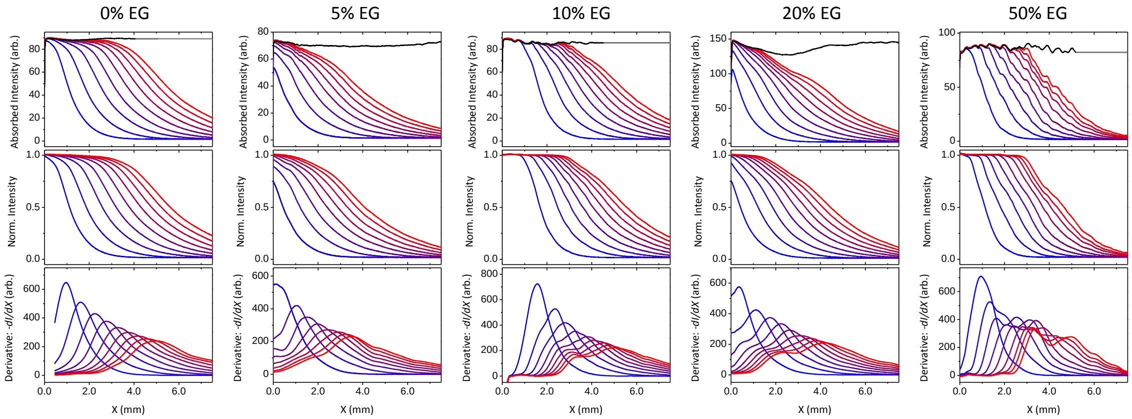

Supplementary Figure 6. The moving front of films prepared from PEDOT:PSS formulations with 0-50 vol% EG content . Top row: Raw data for t = 5 s (blue) to t = 45 s (red), black line represents the absorption intensity of the fully de-doped film over a length of *X*, used for normalization. Middle row: normalized spectra, a value of 1 is the de-doped intensity shown as black line at top. Bottom row: Derivative profiles (A_{n+1}-A_n) of the normalized spectra.

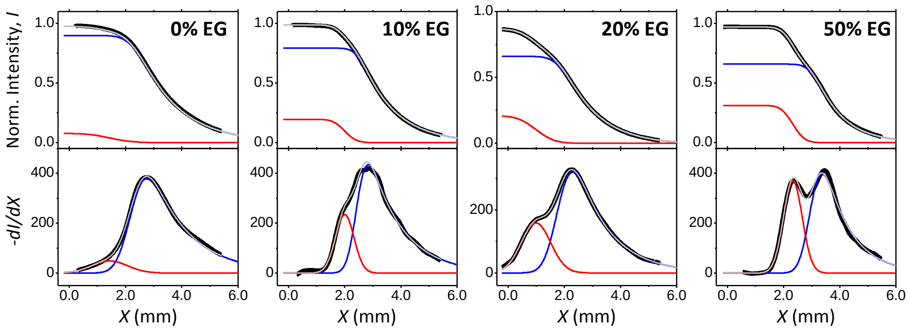

Supplementary Figure 7. Deconvolution of moving front into lagging and leading front components. Black trace is data at time t=20-25 s. In the derivative, gray line is fit to the sum of a Gaussian function (red line, lagging front), and an exponential Gaussian (blue line, leading front). This fit is then integrated to show the deconvolution of the profile.

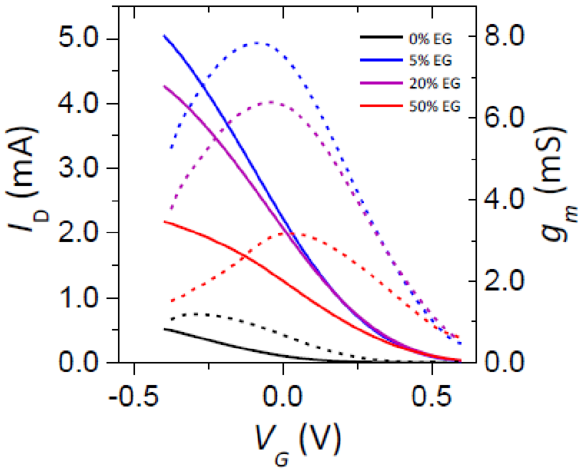

Supplementary Figure 8. Representative device data from formulations of varying EG content. Transfer, I_D-V_G, electrical characteristics (solid) and corresponding transconductance curves (dotted) for OECTs of varying EG formulation content. Black (0 vol% EG), blue (5 vol% EG), purple (20 vol% EG), and red (50 vol% EG). All devices are operated at V_D=-0.6 V, with nominal device dimensions of W=50 µm, L=50 µm; these particular devices have thicknesses of 390 nm (0 vol% EG), 174 nm (5 vol% EG), 208 nm (20 vol% EG) and 184 nm (50 vol% EG).

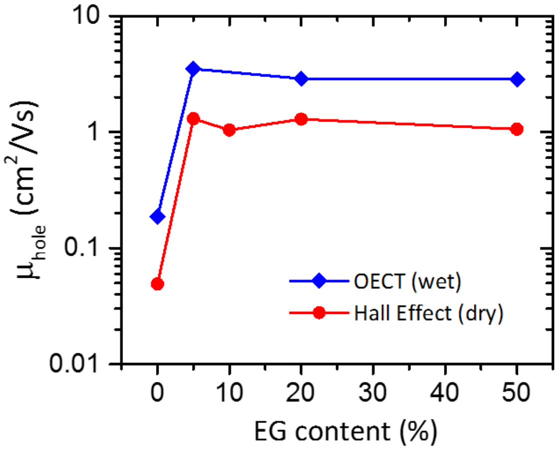

Supplementary Figure 9. Comparison of mobilities of dry and hydrated PEDOT:PSS films cast from varying EG content in dispersion. Dry mobility estimated from Hall Effect measurements are shown in red, mobility from operating, hydrated OECTs are in blue.

Supplementary Notes

Supplementary Note 1

Conversion of Peak Area to RMS concentration fluctuation Δx_{12} of the mesoscale domains can be calculated from the two-phase Porod Invariant formalism:

$$\frac{TSI\prime}{t \cdot e^{-OD}} = |\Delta\rho_{12}|^2 \phi_1 \phi_2 \quad (1)$$

Here, $TSI' = (\text{Peak Area}) \cdot (Detector\ Calibration)$, t is film thickness, e^{-OD} is film and SiN window transmission, ϕ_1 is the volume fraction for domain 1, and $|\Delta\rho_{12}|^2 = \left(\frac{2\pi}{\lambda^2}\right)^2 |\Delta n_{AB}|^2 \cdot |\Delta x_{12}|^2$, where n_A is the index of refraction for material A, and $\Delta\eta_{12}$ is the fluctuation in concentration of material A (or B) between domains 1 and 2. Detector calibration represents the responsivity of the CCD for scattering signal and the photodiode for incident beam intensity. These were calibrated together by measuring the scattering intensity of a PS-*b*-PMMA block-copolymer sample where domains are well organized, pure, and the domain volume fraction is well-controlled. The concentration fluctuation Δx_{12} in Supplementary Figure 3e is finally converted into individual domain concentrations presented in Figure 2c by constraining the average concentration to be equal to the compositions measured in Supplementary Figure 5b.
